# Severity and outcomes of influenza-related pneumonia in type A and B strains in China, 2013–2019

**DOI:** 10.1186/s40249-020-00655-w

**Published:** 2020-04-22

**Authors:** Liang Chen, Xiu-Di Han, Yan-Li Li, Chun-Xiao Zhang, Xi-Qian Xing

**Affiliations:** 1 Department of Infectious Diseases, Beijing Jishuitan Hospital, 4th Medical College of Peking University, Beijing, China; 2 Department of Pulmonary and Critical Care Medicine, Qingdao Municipal Hospital, Qingdao City, Shandong Province China; 3 Department of Infectious Diseases and Clinical Microbiology, Beijing Chao-Yang Hospital, Capital Medical University, Beijing, China; 4 Department of Pulmonary and Critical Care Medicine, Beijing Huimin Hospital, Beijing, China; 5 Department of Pulmonary and Critical Care Medicine, the 2nd People’s Hospital of Yunnan Province, Kunming City, Yunnan Province China

**Keywords:** Influenza, Pneumonia, Virus type, Clinical outcome

## Abstract

**Background:**

Inconsistencies exist regarding the severity of illness caused by different influenza strains. The aim of this study was to compare the clinical outcomes of hospitalized adults and adolescents with influenza-related pneumonia (Flu-p) from type A and type B strains in China.

**Methods:**

We retrospectively reviewed data from Flu-p patients in five hospitals in China from January 2013 to May 2019. Multivariate logistic and Cox regression models were used to assess the effects of influenza virus subtypes on clinical outcomes, and to explore the risk factors of 30-day mortality for Flu-p patients.

**Results:**

In total, 963 laboratory-confirmed influenza A-related pneumonia (FluA-p) and 386 influenza B-related pneumonia (FluB-p) patients were included. Upon adjustment for confounders, multivariate logistic regression models showed that FluA-p was associated with an increased risk of invasive ventilation (adjusted odds ratio [*aOR*]: 3.824, 95% confidence interval [*CI*]: 2.279–6.414; *P* <  0.001), admittance to intensive care unit (*aOR*: 1.630, *95% CI*: 1.074–2.473, *P* = 0.022) and 30-day mortality (*aOR*: 2.427, *95% CI*: 1.568–3.756, *P* <  0.001) compared to FluB-p. Multivariate Cox regression models confirmed that influenza A virus infection (hazard ratio: 2.637, *95% CI*: 1.134–6.131, *P* = 0.024) was an independent predictor for 30-day mortality in Flu-p patients.

**Conclusions:**

The severity of illness and clinical outcomes of FluA-p patients are more severe than FluB-p. This highlights the importance of identifying the virus strain during the management of severe influenza.

## Background

Influenza is a contagious respiratory disease that is widespread across the globe. Despite advances in medical technology, influenza causes considerable hospitalizations and mortality [1, 2]. It is estimated that each year, 1 billion cases of symptomatic influenza infection have occurred across the globe, including 3–5 million cases of severe illness and 290 000–650 000 cases of influenza-related respiratory deaths [3]. From 2010 to 2018, approximately 4.3–23 million medical visits, 140 000–960 000 hospitalizations, 18 000–96 000 influenza-related intensive care unit (ICU) admissions and 12 000–79 000 deaths were associated with influenza per year in the United States of America [4]. The disease burden of influenza in Asia is similar to that of western countries [5, 6]. Influenza infection also poses an economic burden. Recent estimates place the economic burden of a moderately severe to severe pandemic at approximately USD 500 billion, or 0.6% of the global income [7]. For these reasons, influenza epidemics are regarded as the greatest threat to the public health in the twenty-first century.

Influenza presents with non-specific symptoms, including sudden onset fever, headache, a sore throat and cough. Kilbourne suggested that the disease features caused by different influenza virus subtypes are clinically indistinguishable [8]. Several studies have examined the hypothesis that the severity of illness caused by influenza is associated with causal virus types. For example, Mosnier & Irving found that the clinical symptoms and outcomes for patients with influenza A and B infections were comparable [9, 10]. Studies by Kaji and colleagues showed that influenza A infection was more severe than influenza B [11]. The outcomes of different studies have been variable in terms of sample size, study settings, populations, and the ability to control potential confounders. Despite inconsistent findings, to understand the differences of the severity and outcomes between specific influenza virus types is of great significance to arrange rational diagnositic testings, carry out prompt antiviral treatment and make other clinical decisions in the management of severe influenza.

Influenza-related pneumonia (Flu-p) is the major kind of severe influenza, which contributes to 20–50% of influenza-related hospitalizations [12]. Here, we conducted a multicenter, retrospective study aimed to evaluate the impact of virus type A and type B on the illness severity and clinical outcomes of immunocompetent, adolescents and adults hospitalized with Flu-p onset in community.

## Methods

### Study design

#### Patient recruitment

We screened hospitalized patients positive for influenza virus RNA at the microbiology labs of five tertiary hospitals in China from 1 January 2013 to 31 May 2019 (Additional file 1). Patients with laboratory-confirmed Flu-p were included. Exclusion criteria were as follows: (i) Aged ≤ 14 years; (ii) not classified as community-onset pneumonia (pneumonia onset ≥ 48 h post-admission and hospitalized within the last 28 days [13]), as it was difficult to determine whether nosocomial pneumonia occurred after the onset the influenza; (iii) it has been reported that the clinical characteristics and outcomes of immunocompromised patients with influenza differ to those of immunocompetent hosts. So, those who are immunocompromised were excluded [14].

#### Disease and treatment definitions

Patients with influenza-related pneumonia were defined during the influenza season and manifested with respiratory symptoms and were positive for influenza virus by reverse-transcription polymerase chain reaction (RT-PCR), together with pulmonary infiltrates on chest radiographs. Early neuraminidase inhibitor (NAI) treatment was defined as any NAI (oseltamivir, zanamivir and peramivir) administered within 48 h of illness onset [15]. Systemic corticosteroid use was defined as at least one dose of any systemic corticosteroid administrated during hospitalization.

#### Data collection

Data were retrospectively collected and included demographic information, underlying diseases (comorbidities are defined in Additional file 1), clinical symptoms, vital signs, laboratory and radiological findings at admission, community-acquired respiratory co-infections (Additional file 1 [16]), clinical management (administration of NAIs, systemic corticosteroids, vasopressor agents, invasive and non-invasive mechanical ventilation) and outcomes (admittance to ICU, length of hospital stay and 30-day mortality). Patients with hospital stays < 30 days were followed up by phone calls to determine survival status.

#### Data analysis

Data were analysed for normality using a Kolmogorov–Smirnov test. Measurement data with a normal distribution are shown as the mean ± standard deviation. Those with a non-normal distribution are expressed as the median. Categorical variables were analyzed using the Chi-square or Fisher’s exact test. Continuous variables were analyzed using a Student’s *t* test or Mann-Whitney *U* test. *P*-values ≤ 0.05 were considered significant. All probability tests were two-tailed.

To evaluate the impact of influenza virus subtypes on illness severity and clinical outcomes (invasive ventilation, admittance to ICU and 30-day mortality) in Flu-p patients, multivariate logistic regression models were established following adjustment for age, sex, comorbidities, pregnancy, obesity, smoking history, early NAI therapy, systemic corticosteroid use, and coinfection with other pathogens. These risk factors were previously shown to be associated with the clinical outcomes of influenza patients and served as confounders [15].

According to the survival status at 30 days post-admission, patients were divided into survival and deceased groups. Baseline characteristics of these patients were then compared. To identify the risk factors for 30-day mortality in Flu-p patients, variables with *P*-values < 0.1 in univariate analysis and influenza virus type A were entered into the multivariate Cox regression analysis. All analyses were performed using Statistical Package for Social Science 22.0 (SPSS, Chicago, IL, USA).

## Results

### Screening process

**Fig. 1:**
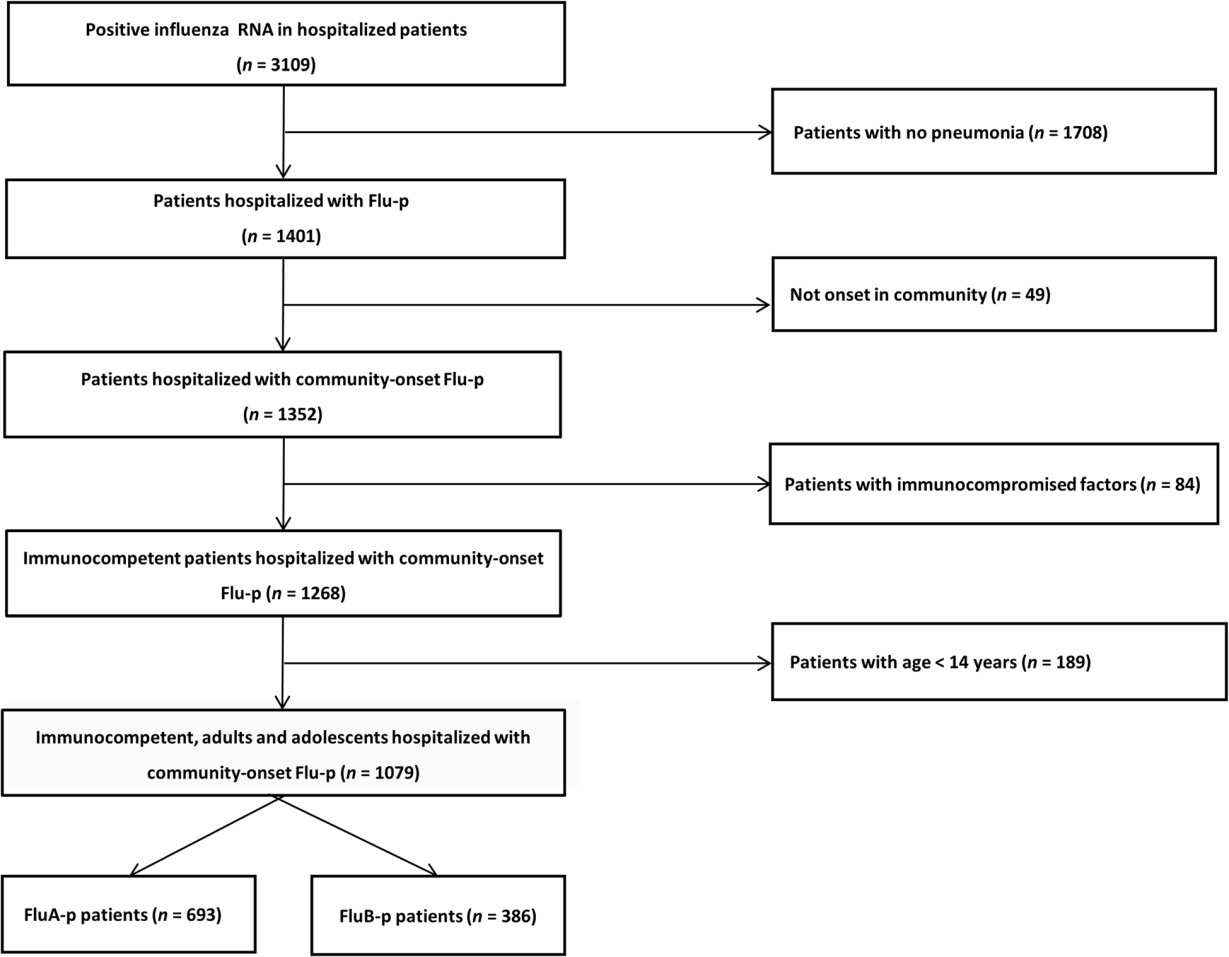
Screening algorithm of patients hospitalized with Flu-p in China, 2013–2019. 3190 patients with influenza RNA positive were screened. Totally, 693 laboratory-confirmed FluA-p patients and 386 FluB-p patients were included into the study

We screened 3190 patients that were influenza RNA positive. A total of 693 laboratory-confirmed FluA-p patients and 386 FluB-p patients were included (Fig. 1). Amongst the FluA-p patients, 38.1% (264/693) were infected with A (H1N1) pmd09 and 11.0% (76/693) were infected with A (H3N2). In total, 50.9% (353/693) of patients were infected with an unclassified subtype.

### Clinical characteristics of flu-p patients

The median age of the Flu-p patients was 61.0 years old. Males accounted for 54.1% (584/1079) of Flu-p patients. More than 50% had at least one underlying disease, including cardiovascular disease 24.0% (259/1079), diabetes mellitus 11.8% (127/1079) and cerebrovascular disease 9.0% (97/1079). In total, 29% (313/1079) of patients had a history of smoking. Axillary temperatures ≥ 38 °C (75.4%, 814/1079) and cough/sputum (98.2%, 1060/1079) were the most common symptoms. Confusion and respiratory rates ≥ 30 beats/min were observed in 13.9% (150/1079) and 13.5% (146/1079) of patients, respectively. Only 1.4% (15/1079) of patients showed systolic blood pressure < 90 mmHg at admission. In total, 46.8% (480/1025) of patients had PO_2_/FiO_2_ < 300 mmHg and 73.6% (794/1079) showed multilobar infiltrates on chest radiology (Table 1).

**Table 1:** The comparison of demographic and clinical characteristics between patients hospitalized with FluA-p and FluB-p in China, 2013–2019

Variable	Total (*n* = 1079)	FluA-p (*n* = 693)	FluB-p (*n* = 386)	*P*-value
Age (years, median, IQR)	61.0 (49.0–78.0)	61.0 (36.0–73.0)	67.0 (55.0–80.0)	***<*0.001**
Male (*n*, %)	584 (54.1)	461 (66.5)	123 (31.9)	***<*0.001**
Days from disease onset to admission (median, IQR)	3.0 (2.0–4.0)	3.0 (2.0–4.0)	3.0 (2.0–4.3)	0.082
Comorbidities (*n*, %)				
Cardiovascular disease	259 (24.0)	136 (19.6)	123 (31.9)	***<*0.001**
Cerebrovascular disease	97 (9.0)	72 (10.4)	25 (6.5)	**0.031**
Diabetes Mellitus	127 (11.8)	92 (13.3)	35 (9.1)	**0.040**
COPD	91 (8.4)	40 (5.8)	51 (13.2)	***<*0.001**
Asthma	33 (3.0)	19 (2.7)	14 (3.6)	0.418
Chronic kidney disease	30 (2.8)	16 (2.3)	14 (3.6)	0.207
Solid Malignant tumor	24 (2.2)	16 (2.3)	8 (2.1)	0.801
Obesity	76 (7.0)	48 (6.9)	28 (7.3)	0.840
Pregnancy	8 (0.7)	8 (1.2)	0 (0.0)	0.080
Smoking history	313 (29.0)	243 (35.1)	70 (18.1)	***<*0.001**
Baseline clinical and radiologic features (*n*, %)				
Axillary temperature ≥ 38 °C	814 (75.4)	661 (95.4)	153 (39.6)	***<*0.001**
Cough/sputum	1060 (98.2)	679 (98.0)	381 (98.7)	0.386
Confusion	150 (13.9)	32 (4.6)	118 (30.6)	***<*0.001**
Respiratory rates ≥ 30 beats/min	146 (13.5)	121 (17.5)	25 (6.5)	***<*0.001**
SBP < 90 mmHg	15 (1.4)	8 (1.2)	7 (1.8)	0.375
Leukocytes > 10 × 10^9^/L	283 (26.2)	118 (17.0)	165 (42.7)	***<*0.001**
Lymphocytes < 0.8 × 10^9^/L	480/1063 (45.2)	299/677 (44.2)	181 (46.9)	0.390
HB < 100 g/L	240 (22.2)	69 (10.0)	171 (44.3)	***<*0.001**
ALB < 35 g/L	187/1025 (18.2)	58/639 (9.1)	129 (33.4)	***<*0.001**
BUN > 7 mmol/L	446/1071 (41.6)	183/685 (26.7)	263 (68.1)	***<*0.001**
Arterial pH < 7.35	171/1025 (16.7)	120/639 (18.8)	51 (12.7)	**0.021**
PO_2_/FiO_2_ < 300 mmHg	480/1025 (46.8)	340/639 (53.2)	140 (36.3)	***<*0.001**
Multilobar infiltrates	794 (73.6)	546 (78.8)	248 (64.2)	***<*0.001**
Coinfections (*n*, %)	367 (34.0)	265 (38.2)	102 (26.4)	***<*0.001**

*IQR* Interquartile range, *COPD* Chronic obstructive pulmonary disease; *SBP* Systolic blood pressure, *HB* Haemoglobin, *ALB* Albumin, *BUN* Blood urea nitrogen, *pH* Hydrogen ion index, *PO*_*2*_*/FiO*_*2*_ Arterial pressure of oxygen/fraction of inspiration oxygen

Other community-acquired pathogens were present in 34.0% (367/1079) of Flu-p patients. *Klebsiella pneumoniae* (31.6%, 116/367) was the most common, followed by *Streptococcus pneumoniae* (29.7%, 109/367) and *Staphylococcus aureus* (19.3%, 71/367) (Additional File 1).

The clinical management and outcomes of Flu-p patients are shown in Table 2. All received antibiotics and NAI, with early NAI administrated to 35.7% (385/1079) of patients. In total, 24.3% (262/1079) of patients received systemic corticosteroids during hospitalization, whilst 23.1% (249/1079), 24.6% (265/1079) and 4.9% (53/1079) developed respiratory failure, heart failure and septic shock, respectively. In total, 17.9% (193/1079) of patients received invasive ventilation and 22.4% (242/1079) were admitted to the ICU. The 30-day mortality rates were 19.3% (208/1079).

**Table 2:** The comparison of clinical management and outcomes between patients hospitalized with FluA-p and FluB-p in China, 2013–2019

Variable	Total (*n* = 1079)	FluA-p (*n* = 693)	FluB-p (*n* = 386)	*P*-value
Early NAI therapy (*n*, %)	385 (35.7)	232 (33.5)	153 (39.6)	**0.043**
Systemic corticosteroid use during hospitalization (*n*, %)	262 (24.3)	132 (19.0)	130 (33.7)	**< 0.001**
Complications during hospitalization				
Respiratory failure	249 (23.1)	167 (24.1)	82 (21.2)	0.286
Heart failure	265 (24.6)	147 (21.2)	118 (30.6)	**0.001**
Septic shock	53 (4.9)	36 (5.2)	17 (4.4)	0.565
Acute renal failure	39 (3.6)	27 (3.9)	12 (3.1)	0.507
Bloodstream infection	9 (0.8)	8 (1.2)	1 (0.3)	0.121
Noninvasive ventilation (*n*, %)	279 (25.9)	159 (22.9)	120 (31.1)	**0.003**
Invasive ventilation (*n*, %)	193 (17.9)	158 (22.8)	35 (9.1)	**< 0.001**
Vasopressor use (*n*, %)	40 (3.7)	27 (3.9)	13 (3.4)	0.660
Admittance to ICU (*n*, %)	242 (22.4)	176 (25.4)	66 (17.1)	**0.001**
Length of stay in hospital (days, median, IQR)	10.0 (8.0–14.0)	12.0 (7.0–14.5)	10.0 (8.0–17.0)	**< 0.001**
30-day mortality (*n*, %)	208 (19.3)	136 (19.6)	72 (18.7)	0.698

*NAI* neuraminidase inhibitor, *ICU* intensive care unit; IQR: Interquartile range

### Comparison of patients hospitalized with FluA-p and FluB-p

Compared to patients with FluB-p, FluA-p patients were younger and predominantly male. In FluA-p patients, cerebrovascular disease, diabetes mellitus and smoking history were frequent, whilst cardiovascular disease was less common. FluA-p patients more frequently showed axillary temperatures ≥ 38 °C, confusion, arterial hydrogen ion index (pH) < 7.35, PO_2_/FiO_2_ < 300 mmHg and multilobar infiltrates compared to FluB-p patients. More FluA-p patients had coinfections (Table 1).

A larger number of FluB-p patients received early NAI, systemic corticosteroid therapy and developed complications such as heart failure during hospitalization. Invasive ventilation was more frequent in FluA-p patients. The length of stay in hospital was significantly longer in FluA-p patients compared to FluB-p patients. The 30-day mortality rates were similar between the two groups (Table 2).

### Impact of virus type on the severity of illness and clinical outcomes of flu-p patients

Univariate logistic analysis showed that influenza A virus infection was associated with an increased risk of invasive ventilation (*OR:* 2.811, 95% *CI:* 1.905–4.167, *P* <  0.001) and admittance to the ICU (*OR:* 1.651, 95% *CI:* 1.204–1.204, *P* = 0.002), but did not correlate with 30-day mortality (*OR:* 1.065, 95% *CI:* 0.775–1.463, *P* = 0.698) in Flu-p patients (Table 3).

**Table 3:** The impact of influenza virus type A on the illness severity and outcomes of patients hospitalized with Flu-p in China, 2013–2019

Variable	Univariate logistic analysis		Multivariate logistic analysis	
	*OR* (95% *CI*)	*P-*value	*a*OR* (95% *CI*)	*P-*value
Invasive ventilation	2.811 (1.905–4.167)	< 0.001	3.824 (2.279–6.414)	< 0.001
Admittance to ICU	1.651 (1.204–1.204)	0.002	1.630 (1.074–2.473)	0.022
30-day mortality	1.065 (0.775–1.463)	0.698	2.427 (1.568–3.756)	< 0.001

*OR* Odd ratio, *CI* Confidence interval, *ICU* Intensive care unit. *: adjusted for age, sex, comorbidities, pregnancy, obesity, smoking history, early NAI treatment and systemic corticosteroid, and coinfection with other pathogens

Following adjustment for age, sex, comorbidities, pregnancy, obesity, smoking history, early NAI treatment and systemic corticosteroid use, and coinfections, multivariate logistic regression models revealed that influenza A virus infection was associated with an increased risk of invasive ventilation (*OR:* 3.824, 95% *CI:* 2.279–6.414, *P* <  0.001), ICU admission (*OR:* 1.630, 95% *CI:* 1.074–2.473, *P* = 0.022) and 30-day mortality (*OR:* 2.427, 95% *CI:* 1.568–3.756, *P* <  0.001) in Flu-p patients (Table 3).

**Fig. 2:**
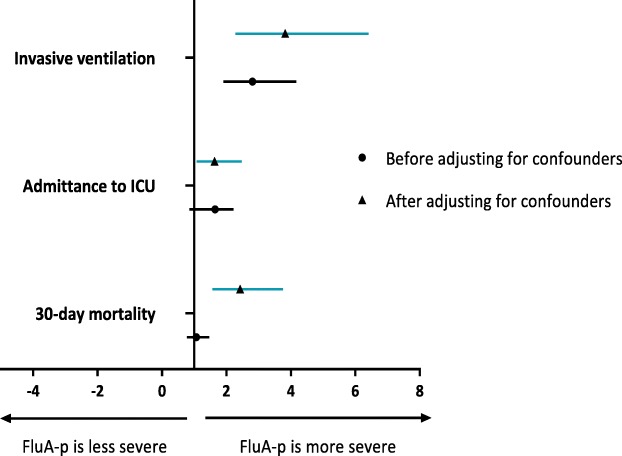
Forrest plot of the impact of influenza virus type on the illness severity and outcomes of patients hospitalized with Flu-p in China, 2013–2019. Before adjusting for confounders, influenza A virus infection was associated with an increased risks of invasive ventilation and admittance to intensive care unit (ICU), but did not correlate with 30-day mortality. After adjusting for confounders, influenza A virus infection was associated with an increased risks of invasive ventilation, ICU admission and 30-day mortality in Flu-p patients

The forrest plots of the impact of influenza virus A on invasive ventilation, admittance to the ICU and 30-day mortality in Flu-p patients after and prior to adjusting for confounders are shown in Fig. 2.

### Risk factors for 30-day mortality in flu-p patients

Logistic regression analysis allowed us to explore the factors for 30-day mortality in Flu-p patients. All potential factors screened in the univariate analysis with *P* <  0.1 and influenza A virus infection were added to the Cox regression model (Additional file 1).

Multivariate Cox regression models confirmed that influenza A virus infection (hazard ratio [*HR*]*:* 2.637, 95% *CI:* 1.134–6.131, *P* = 0.024), age (*HR:* 1.055, 95% *CI:* 1.033–1.077, *P* <  0.001), cardiovascular disease (*HR:* 7.683, 95% *CI:* 3.175–18.58, *P* <  0.001), smoking history (*HR:* 3.137, 95% *CI:* 1.417–7.124, *P* <  0.001), lymphocytes < 0.8 × 10^9^/L (*HR:* 10.473, 95% *CI:* 5.033–21.792, *P* <  0.001), blood urea nitrogen (BUN) > 7 mmol/L (*HR*: 3.170, 95% *CI*: 1.449–6.935, *P* = 0.004) and arterial pH < 7.35 (*HR:* 3.037, 95% *CI:* 1.552–5.945, *P* = 0.001) were independent risk factors for 30-day mortality in Flu-p patients (Table 4).

**Table 4:** The risk factors for 30-day mortality of patients hospitalized with Flu-p in China, 2013–2019

Variable	*P-*value	*aHR* (95% *CI*)
Influenza virus A infection	0.024	2.637 (1.134–6.131)
Age	< 0.001	1.055 (1.033–1.077)
Cardiovascular disease	< 0.001	7.683 (3.175–18.589)
Smoking history	< 0.001	3.137 (1.417–7.124)
Lymphocytes < 0.8 × 10^9^/L	< 0.001	10.473 (5.033–21.792)
BUN > 7 mmol/L	0.004	3.170 (1.449–6.935)
Arterial pH < 7.35	0.001	3.037 (1.552–5.945)

a*HR* adjusted hazard ratio, *CI* Confidence interval, *BUN*: Blood urea nitrogen

**Fig. 3:**
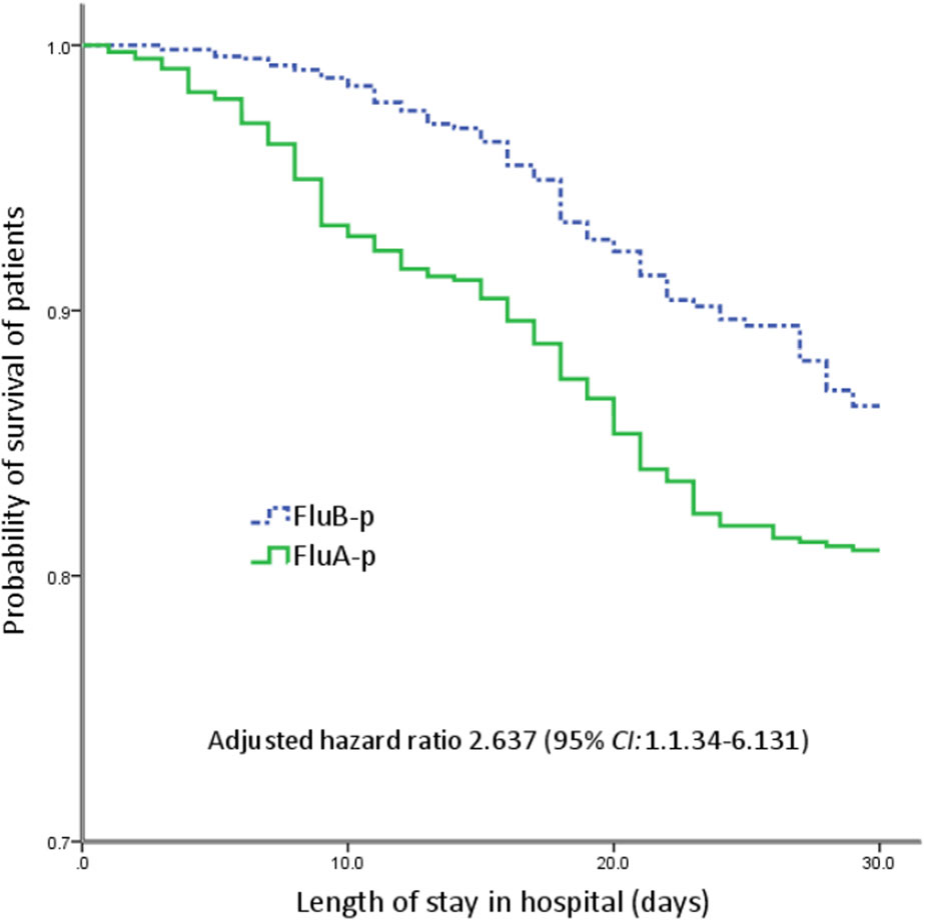
Survival rate of patients hospitalized with FluA-p and FluB-p in China, 2013–2019 (censored at 30 d after admission). The 30-day mortality of FluA patients was higher than that of FluB-p patients after adjusting for confounders

The survival curve shows that the 30-day mortality of FluA patients was higher than that of FluB-p patients after adjusting for confounders (age, cardiovascular disease, chronic kidney disease, smoking history, confusion, lymphocytes < 0.8 × 10^9^/L, hemoglobin < 100 g/L, BUN > 7 mmol/L, arterial pH < 7.35, PO_2_/FiO_2_ < 300 mmHg, coinfections and systemic corticosteroid use) (Fig. 3).

## Discussion

This large-sample cohort study showed that illness severity and clinical outcomes were poorer in patients hospitalized with FluA-p as opposed to FluB-p after adjusting for potential confounders, suggesting a direct impact of influenza virus types on the characteristics and outcomes of influenza related pneumonia.

In this study, the 30-day mortality was 19.6%, which was accordant with the 5–50% reported in previous reports [17–19]. The median age was 61.0 years and over 50% of patients had co-morbidities, delayed NAI therapy and systemic corticosteroid (70 and 25% of patients respectively), which may explain the high mortality rates. The proportion of patients requiring invasive ventilation and ICU admission were higher for FluB-p patients. Although the death rates between the two groups were comparable. Significant differences in the 30-day mortality were observed after controlling for confounders.

Our data were consistent with Wang et al. [20] that included 369 patients with flu A infection and 205 patients with flu B infection. After adjustment for age, sex, heart disease, malignancies and time from illness onset to antiviral treatment, logistic regression models showed a higher probability of clinical improvement (*HR:* 1.266, 95% *CI:* 1.019–1.573) and weaning oxygen supplementation (*HR:* 1.285, 95% *CI:* 1.030–1.603) in flu B patients. The in-hospital mortality of flu A patients was marginally higher than flu B patients (11.4% vs 6.8%; *P* = 0.078), which might be due to the relatively small number of deaths (56 in total). Similarly, Chaves and colleagues [21] performed a retrospective study using population-based influenza hospitalization surveillance data. They found that A (H1N1) pdm09 infection was an independent predictor for illness severity both in children (*aOR:* 2.19, 95% *CI:* 1.11–4.33) and adults (*aOR:* 2.21, 95% *CI:* 1.66–2.943) compared to flu B infection.

In ferret models, A (H1N1) pdm09 strains led to more severe clinical symptoms and histopathology, followed by A (H3N2) strains, whilst Flu B strains had a milder illness [22]. Although the specific pathogenesis governing these effects has not been elucidated, some mechanisms have been postulated. Hemagglutinin (HA) of influenza B virus strains is heavily glycosylated [23]. Since glycosylated HA binds collagenous lectins in lung surfactants, it is easily cleared from the lungs. HA of human influenza B viruses also preferentially bind to α-2,6-linked sialic acids present in the human upper respiratory tract, whilst A (H1N1) pdm09 viruses bind both α-2,6-linked and α-2,3-linked sialic acids [24]. Influenza B viruses are therefore restricted to the upper respiratory tract, whilst A (H1N1) pdm09 viruses are more prevalent in the lower respiratory tract [25]. Compared to influenza A viruses, influenza B has lower receptor-binding affinity due to the presence of a Phe-95 versus a Tyr-98 in the HA protein, resulting in a loss of hydrogen bonds [26]. The innate IFN response is also more rapidly initiated following influenza B as opposed to influenza A virus infection. This leads to more rapid viral clearance and lower viral titers [27]. In vivo, both influenza A and B viruses downregulate the surface expression of major histocompatibility complex-I (MHC-I). A more pronounced reduction in surface MHC-I expression was observed in influenza B patients, leading to milder immunologic reactions, followed by significantly lower levels of inflammatory cytokines and lung tissue injury [28].

A prospective study from France et al. [29] that included 556 patients with influenza, of which 30% had pneumonia, showed that the admittance to the ICU, not the virus type, was the main risk factor for death. They further confirmed that prior chronic respiratory disease was associated with ICU admission in multivariate logistic regression models. The proportion of chronic respiratory disease patients was significantly higher in flu A compared to flu B patients. However, the association of virus types with ICU admission were not assessed.

Several studies have compared the mortality rates between patients according to virus type, but many failed to control for confounders [9, 10, 30–32]. Recently, a systematic literature review suggested the A (H1N1) pdm09 during the post-pandemic period was more related to poor outcomes (secondary bacterial pneumonia, ICU admission, and death) than influenza B viruses [33].

To our knowledge, this is the first real-word cohort study (with a large population of adolescents and adults admitted to general hospital wards or ICUs) that focused on the association of influenza viruses types with illness severity and clinical outcomes of laboratory-confirmed influenza-related pneumonia patients. Methods were taken to reduce selection bias and control confounders, but some limitations should be noted. First, due to the retrospective nature of the study, potential selection bias may have influenced the data. For example, during each influenza season, patients with influenza-like illness (such as fever, sore throat or cough) were assessed through influenza RNA tests by the subjective judgement of attending physicians in the five hospitals. It was possible that more severe (or milder) patients were tested for influenza. Not all respiratory cases were eligible for swabbing and some selection bias occurred. Secondly, due to the retrospective design, the impact of vaccination on disease severity could not be evaluated, and the inclusion of incomplete data may have lowered the accuracy of our results. Thirdly, there is evidence of different severities of influenza A virus subtypes [11, 32]. However, over 50% of patients were not tested for subtypes in our study. Further work is required to compare the clinical features according to subtype. Finally, our study population were immunocompetent, adolescent and adult hospitalized patients. The conclusions should be assessed prudently prior to similar assessments in immunocompromised patients, pediatrics and outpatients.

## Conclusions

The clinical outcomes of FluA-p are worse than FluB-p, highlighting the importance of influenza virus strain testing in the management of severe influenza. As influenza A virus infection is a predictor for poor outcomes in patients with influenza-related pneumonia, regardless of their ages and chronic underlying conditions, the clinicians should pay more attention to patients with FluA-p. Also, it suggests the priority of vaccination covered influenza virus type A strains in certain populations is rational.

## Supplementary information

**Additional file 1: Appendix 1.** Details of participating centers. **Appendix 2.** Definition of underlying diseases of patients hospitalized with Flu-p in China, 2013–2019. Definition of microbiological criteria of coinfected with other pathogens in patients hospitalized with Flu-p in China, 2013–2019. **Appendix 3.** Coinfection with other community-acquired pathogens in patients hospitalized with Flu-p in China, 2013–2019. **Appendix 4** Univariate analysis on risk factors for 30-day mortality of patients hospitalized with Flu-p in China, 2013–2019.

## Data Availability

All data generated or analysed during this study are included in this published article and supplementary information.

## Abbreviations

BUN: Blood urea nitrogen
*CI*: Interval confidence
Flu-p: Influenza-related pneumonia
HR: Hazard ratio
ICU: Intensive care unit
IQR: Interquartile range
NAI: Neuraminidase inhibitor
*OR*: Odds ratio
pH: Hydrogen ion index
pO_2_/FiO_2_: Arterial pressure of oxygen/fraction of inspiration oxygen
RCT: Randomized controlled trial
RT-PCR: Reverse transcription polymerase chain reaction

## References

[1] Thommes EW, Kruse M, Kohli M, Sharma R, Noorduyn SG (2017). Review of seasonal influenza in Canada: burden of disease and the cost-effectiveness of quadrivalent inactivated influenza vaccines. Hum Vaccin Immunother.

[2] Lozano R, Naghavi M, Foreman K, Lim S, Shibuya K, Aboyans V (2012). Global and regional mortality from 235 causes of death for 20 age groups in 1990 and 2010: a systematic analysis for the global burden of disease study 2010. Lancet..

[3] Ly S, Arashiro T, Ieng V, Tsuyuoka R, Parry A, Horwood P (2017). Establishing seasonal and alert influenza thresholds in Cambodia using the WHO method: implications for effective utilization of influenza surveillance in the tropics and subtropics. Western Pac Surveill Response J.

[4] Reed C, Chaves SS, Daily Kirley P, Emerson R, Aragon D, Hancock EB (2015). Estimating influenza disease burden from population-based surveillance data in the United States. PLoS One.

[5] Leo YS, Lye DC, Chow A (2009). Influenza in the tropics. Lancet Infect Dis.

[6] Feng L, Shay DK, Jiang Y, Zhou H, Chen X, Zheng Y (2012). Influenza-associated mortality in temperate and subtropical Chinese cities, 2003-2008. Bull World Health Organ.

[7] Fan VY, Jamison DT, Summers LH (2018). Pandemic risk: how large are the expected losses?. Bull World Health Org.

[8] Kilbourne ED (2006). Influenza pandemics of the 20th century. Emerg Infect Dis.

[9] Mosnier A, Caini S, Daviaud I, Nauleau E, Bui TT, Debost E (2015). Clinical characteristics are similar across type a and B influenza virus infections. PLoS One.

[10] Irving SA, Patel DC, Kieke BA, Donahue JG, Vandermause MF, Shay DK (2012). Comparison of clinical features and outcomes of medically attended influenza a and influenza B in a defined population over four seasons: 2004-2005 through 2007-2008. Influenza Other Respir Viruses.

[11] Kaji M, Watanabe A, Aizawa H (2003). Differences in clinical features between influenza a H1N1, a H3N2, and B in adult patients. Respirology.

[12] Fu X, Zhou Y, Wu J, Liu X, Ding C, Huang C (2019). Clinical characteristics and outcomes during a severe influenza season in China during 2017-2018. BMC Infect Dis.

[13] Chen L, Zhou F, Li H, Xing X, Han X, Wang Y (2018). Disease characteristics and management of hospitalised adolescents and adults with community-acquired pneumonia in China: a retrospective multicentre survey. BMJ Open.

[14] Kossyvakis A, Mentis AA, Tryfinopoulou K, Pogka V, Kalliaropoulos A (2017). Antiviral susceptibility profile of influenza a viruses; keep an eye on immunocompromised patients under prolonged treatment. Eur J Clin Microbiol Infect Dis.

[15] Muthuri SG, Venkatesan S, Myles PR, Leonardi-Bee J, Al Khuwaitir TS, Al Mamun A (2014). Effectiveness of neuraminidase inhibitors in reducing mortality in patients admitted to hospital with influenza a H1N1pdm09 virus infection: a meta-analysis of individual participant data. Lancet Respir Med.

[16] Jain S, Self WH, Wunderink RG, Fakhran S, Balk R, Bramley AM (2015). Community-acquired pneumonia requiring hospitalization among U.S. adults. N Engl J Med.

[17] MacIntyre CR, Chughtai AA, Barnes M, Ridda I, Seale H, Toms R (2018). The role of pneumonia and secondary bacterial infection in fatal and serious outcomes of pandemic influenza a(H1N1) pdm09. BMC Infect Dis.

[18] Liu D, Xu J, Yu X, Tong F, Walline J, Fu Y (2019). Clinical characteristics and prognosis of influenza B virus-related hospitalizations in northern China during the 2017-18 influenza season: a multicenter case series. Biomed Res Int.

[19] Sohn CH, Ryoo SM, Yoon JY, Seo DW, Lim KS, Kim SH (2013). Comparison of clinical features and outcomes of hospitalized adult patients with novel influenza a (H1N1) pneumonia and other pneumonia. Acad Emerg Med.

[20] Wang Y, Fan G, Horby P, Hayden F, Li Q, Wu Q (2019). Comparative outcomes of adults hospitalized with seasonal influenza A or B virus infection: Application of the 7-category ordinal scale. Open Forum Infect Dis.

[21] Chaves SS, Aragon D, Bennett N, Cooper T, D'Mello T, Farley M (2013). Patients hospitalized with laboratory-confirmed influenza during the 2010-2011 influenza season: exploring disease severity by virus type and subtype. J Infect Dis.

[22] Huang SS, Banner D, Fang Y, Ng DC, Kanagasabai T, Kelvin DJ (2011). Comparative analyses of pandemic H1N1 and seasonal H1N1, H3N2, and influenza B infections depict distinct clinical pictures in ferrets. PLoS One.

[23] Rogers GN, Paulson JC (1983). Receptor determinants of human and animal influenza virus isolates: differences in receptor specificity of the H3 hemagglutinin based on species of origin. Virology.

[24] Kumlin U, Olofsson S, Dimock K, Arnberg N (2008). Sialic acid tissue distribution and influenza virus tropism. Influenza Other Respir Viruses.

[25] Walther T, Karamanska R, Chan RW, Chan MC, Jia N, Air G (2013). Glycomic analysis of human respiratory tract tissues and correlation with influenza virus infection. PLoS Pathog.

[26] Matrosovich MN, Gambaryan AS, Tuzikov AB, Byramova NE, Mochalova LV, Golbraikh AA (1993). Probing of the receptor-binding sites of the H1 and H3 influenza a and influenza B virus hemagglutinins by synthetic and natural sialosides. Virology..

[27] Österlund P, Strengell M, Sarin LP, Poranen MM, Fagerlund R, Melén K (2012). Incoming influenza a virus evades early host recognition, while influenza B virus induces interferon expression directly upon entry. J Virol.

[28] Koutsakos M, McWilliam HEG, Aktepe TE, Fritzlar S, Illing PT, Mifsud NA (2019). Downregulation of MHC class I expression by influenza a and B viruses. Front Immunol.

[29] Loubet P, Samih-Lenzi N, Galtier F, Vanhems P, Loulergue P, Duval X (2016). Factors associated with poor outcomes among adults hospitalized for influenza in France: a three-year prospective multicenter study. J Clin Virol.

[30] Su S, Chaves SS, Perez A, D'Mello T, Kirley PD, Yousey-Hindes K (2014). Comparing clinical characteristics between hospitalized adults with laboratory-confirmed influenza a and B virus infection. Clin Infect Dis.

[31] Wie SH, So BH, Song JY, Cheong HJ, Seo YB, Choi SH (2013). A comparison of the clinical and epidemiological characteristics of adult patients with laboratory-confirmed influenza a or B during the 2011-2012 influenza season in Korea: a multi-center study. PLoS One.

[32] Ishiguro T, Takayanagi N, Kanauchi T, Uozumi R, Kawate E, Takaku Y (2016). Clinical and radiographic comparison of influenza virus-associated pneumonia among three viral subtypes. Intern Med.

[33] Caini S, Kroneman M, Wiegers T, El Guerche-Séblain C, Paget J (2018). Clinical characteristics and severity of influenza infections by virus type, subtype, and lineage: a systematic literature review. Influenza Other Respir Viruses.

